# The Truth Shall Make Us Free—Third Article

**Published:** 1896-01

**Authors:** J. H. Allen

**Affiliations:** Logansport, Ind.


### THE TRUTH SHALL MAKE US FREE—THIRD ARTICLE.

By J. H. Allen, M. D., Logansport, Ind.

The truth not only makes us free, but it makes us strong. It is the secret of strength. "Our convictions are profound when we are impressed with great and profound truths." It often enables the frailest body to become a conqueror, not for the "bubble, reputation; at the cannon's mouth," nor for political control over his fellow-man, but for a higher result, a victory over himself; letting himself free from his bonds, and guided by the light of truth he comes, as did Hahnemann, upon doors that have been barred from eternity, and opens them revealing more and more of the truth of the similia.

Truth by virtue of its genetic power, must in the end overcome everything that opposes it, as it is an attribute of the Creator Himself. Therefore we have the assurance that if we present anything new to the world that is based upon truth it must stand the test of time; and, although the world for a time rejects it, and uses every means to down it, like Banquo's ghost, "it will not down," but ever presents itself "four-squared to every wind that blows." Some one has said we are "imitators of our ideals;" that is, if we are followers of Socrates we become like Socrates; if followers of Christ we become Christ-like; or if we are followers of Hughes or Dudgeon we rise no higher in the scale of Homœopathy than they do, as a rule, and their conception of its law must be our conception. But if we follow

Hahnemann and his *Organon of Medicine*, we are lifted up into the higher altitudes of truth, and brought face to face with the infinite—face to face with his life-restoring principle, *similia*, the expression of whose action is the image of the Unseen. Yea, this science of Homœopathy leads us up to the edge of the infinite, and enables us to fight our battles with these unseen forces, these principalities and powers of darkness, whose name is legion ; these miasms ; these morbid forces and influences that try to bind or fetter true biological law or drive life from its princely citadel with their legions of foul fiends that environ us.

Truth again leads us to the study of the true phenomena of life, to get a clearer conception and knowledge of its nature, and to the study of the phenomena of all biological law. We are not satisfied with the study of the anatomical man, in which is no life, nor with the study of the dead cell that we have removed for study and set apart for examination, as it is the result or the workmanship of life, a sarcophagus, a debris of life, and we cannot see it when it dwells in its princely mansion, vivified by that God-given principle which we call life. So our investigations are in dead organized matter, instead of with true organized life. They are empty shells or husks, where dwelt the principle that has fled. Overcome are these wonderful life forces by some more potent subversive force ; the gravitational law governing biological life is set aside, then in cometh death and desolation. Alas ! what a debt of gratitude we owe to Hahnemann for this most delightful retrospect of biological law, broadening our minds and our conceptions of law, of life, and of man in general, and his peculiar complex relationship with this world, as well as to all physical law ; a law by which we can compare ourselves with the infinite and measure ourselves with eternity. Homœopathy is the beating of a kind heart through the scheme of things. It cometh like Portia's mercy, gently, and is twice blessed : "It blesseth him that giveth and him that taketh." It is the Christ through the manifestation of a divine law ; a grace-given principle to the sick and pain-suffering physical man. If we have offended the great central Soul of the universe the majesty of Law says,

"Thou must suffer the penalty prescribed," but the similia power comes to us in our extremity and need, and sets aside the penalty by satisfying the demands of the law through its vicarious power in suspending the action of all subversive forces, acting subversively on biological law.

Truth has no rents, no tears or patches, but is a whole garment throughout. It is the true orbital band around which all true thought must revolve, and the time must come when it will belt the universe and be to all men the *Mene Mene Tekel Upharsin* of all law, of all philosophy, of all true science, and the zeal of its fire will burn out the false and all that maketh a lie, and when it must openly and boldly expunge the unclean thing and come out unsullied and without a spot upon its garments. The world frowns upon it, but it shines all the brighter, and, like the diamond, throws out its light from every angle. The influence of similia steals over us so gently and through the law its influence is quietly impressed upon our disturbed life forces, and the change comes by virtue of the law being fulfilled in itself; and through its creative power it creates health, and health creates bone and muscles and nerve force and power of will and thought and mind; and a healthy mind and body bring joy and peace and happiness and contentment, even all the attributes of a normally restored vital force.

This is no chance world, but a world of method and principle and immutable law; and all nature is a standing protest against empiricists and chance workers, and the absurdity of trying to secure true effects other than through the retrograde process of law, without doing injury or violence to the sick one, in readjusting the life forces that are disturbed and setting them again in working harmony and bringing them into true relationship with their surrounding media. Health is a perfect poise, an absolute adjustment of the forces of the ego to all other forces, be they what they may. On the one hand Nature seems to care nothing for us, yet on the other she fosters us: so we seem to be constantly under stress, under which we seem to rise or fall, keeping up the great equilibrium of life.

*That great pulse in Nature that beats against the barriers of*

*materialism is the pulse of love, and with each throb comes a new genesis, new life; in its multitudinous forms stimulating all biological law to new energy and to greater power in its transference of unorganized matter to organized life.*

*Man is a divinity in his earthly disguise, when guided by the power of truth, which glorifies and clothes him in the true light of science; when Alpine-like he climbs; his vision widening with his widening mind; drinking in the largeness of infinity, and gracefully submitting to every law and every principle that the truth may present to him for the furtherance of his knowledge; the visible being his starting point and the invisible his goal.* From such a mind Nature cannot keep hid her secrets; no hieroglyphics so old but what he can read; no language so dead but what he can resurrect, and they speak to him in tongues of fire. Even the universe, with all its vastness, with its thousand-fold complexity of forces; yea, the truth of Homœopathy gives us power to escape out of the "hear-says" of uncertainty in our study of life, and we are brought face to face with the true Shekinah in man; and the secret of the *I* is made manifest; and this Triune temple of the universe, which, "when we touch," says Carlyle, "we touch heaven;" this human body of ours, "this miracle of miracles, this mystery of the living God." Through the truths of Homœopathy we are brought to the knowledge of the prenomena of life, when under the law of true harmonic force and also when under the power of false or subversive forces. It reveals to us the antipathic schools of medicine in their true light, with their wrappings and bandages of tradition, with their eyes fixed only on materialism; with no law, no God in their man or in their science. "Icy, regular, splendidly null" and void of all that spirit of truth which we find in that great book, *The Organon*, which reveals man as that mysterious force in the centre of all forces. Ah! listen to the words of Hahnemann, his words of wisdom and of healing, who spent his life in revealing the truth, in demonstrating law, in unmasking hypocrisy in high places; a truth which enables us to look into the mysteries of life with eyes of the soul, and to put ourselves in true relationship with Nature and her powers.

